# Non-invasive interactive neurostimulation (InterX ™) reduces acute pain in patients following total knee replacement surgery: a randomised, controlled trial

**DOI:** 10.1186/1749-799X-6-45

**Published:** 2011-08-24

**Authors:** Ashok K Nigam, Drena M Taylor, Zulia Valeyeva

**Affiliations:** 1 Prince Philip Hospital, Carmarthenshire NHS Trust, Mawr Dafen, Llanelli, UK; 2 InterX Clinic Cheltenham, Maple House, Bayshill Rd, Cheltenham, Glouc, UK

## Abstract

**Background:**

*Adequate post-operative pain relief following total knee replacement (TKR) is very important to optimal post-operative recovery. Faster mobilisation and rehabilitation ultimately results in optimum recovery outcomes, but pain is often the limiting factor. This study evaluates the potential clinical benefit of the InterX neurostimulation device on pain reduction and rehabilitative outcome*.

**Methods:**

A clinical trial under the Hywel Dda Clinical Audit Committee to validate the clinical benefit of Non-invasive Interactive Neurostimulation (NIN) therapy using the InterX device was performed in patients undergoing TKR. 61 patients were randomised to treatment groups in blocks of two from the Theatre Operation List. The control group received the standard hospital course of pain medication and rehabilitation twice daily for 3 post-op days. The experimental group received 8 sessions of NIN therapy over 3 post-op days in addition to the standard course received by the Control group. Pain and range of motion were collected as the primary study measures.

**Results:**

Sixty one subjects were enrolled and randomised, but 2 subjects (one/group) were excluded due to missing data at Baseline/Final; one subject in the InterX group was excluded due to pre-existing rheumatoid pain conditions confounding the analysis.

The experimental group pre- to post-session Verbal Rating Scale for pain (VRS) showed that NIN therapy consistently reduced the pain scores by a mean of 2.3 points (SE 0.11). The NIN pre-treatment score at Final was used for the primary ANCOVA comparison, demonstrating a significantly greater cumulative treatment effect of a mean 2.2 (SE 0.49) points pain reduction (p = 0.002). Control subjects only experienced a mean 0.34 (SE 0.49) point decrease in pain. Ninety degrees ROM was required to discharge the patient and this was attained as an average despite the greater Baseline deficit in the InterX group. Eight control patients and three experimental patients did not achieve this ROM.

**Conclusions:**

The results clearly demonstrated the clinical benefit of NIN therapy as a supplement to the standard rehabilitation protocol. The subjects receiving InterX fared significantly better clinically. Within a relatively short 3-day period of time, patients in the experimental group obtained the necessary ROM for discharge and did it experiencing lower levels of pain than those in the control group.

## Background

Adequate post-operative pain relief following total knee replacement (TKR) is very important to optimal post-operative recovery[1]. The faster that mobilisation and rehabilitation can progress, the better the ultimate outcome will be[2]. Adequate pain control postoperatively should allow earlier patient mobilisation with the aim of increasing strength and proprioception and decreasing the incidence of the development of thromboembolism, however side effects of pain medication have been shown to increase the incidence of thromboembolism[3]. In complicated cases, prolonged immobility due to pain can cause the development of muscular contractures or atrophy that eventually cause the development of long term functional impairments[4]. Unfortunately, there are very few pain management options available that can provide a treatment that is both non-invasive and without side effects.

Current pharmaceutical pain relief options for TKR pain have limitations due to associated side effects, often requiring additional treatment for them[5]. Side-effects of the opiate pain medication include lethargy, sedation, respiratory depression, nausea, vomiting, numbness, weakness, urinary retention, hypotension[6] and digestive discomfort, including gastroparesis and constipation[7]. Opioids may also alter mood negatively and/or induce euphoria. The side effects of non-steroidal anti-inflammatory agents (NSAIDS) include gastric upset, sometimes predisposing symptoms leading to peptic ulcers. COX-2 inhibitors have been found to increase risk of heart attack while overdoses can lead to liver damage[8]. Navigating these side effects amongst the co-morbidities and potential drug interactions with concurrent medications in the elderly population is typically problematic.

Historically, electrical stimulation modalities, such as transcutaneous electrical nerve stimulation (TENS), have been used to manage pain and facilitate recovery from various traumatic conditions[9-12]. In general, the drawbacks of using TENS for this application is that the devices use non-specific current dispersed through predominantly large electrode pads, the amplitude of the stimulation is limited by the risk of muscle contraction and the current density is limited by the recommended safe minimum size of the electrodes[13]. A conductive medium (gel), either separately applied or as part of a pre-gelled electrode, is also needed to protect the patient from uncomfortable variations in current that are caused as the tissue responds to the stimulation. Research has shown mixed results in the post-surgical application of TENS. The Bandolier, evidence-based health care web site highlighted a systematic review of TENS and stated: "Clinical bottom line: TENS is not effective in the relief of post-operative pain. Patients should be offered effective methods of pain relief" (Bandolier, 2000). Conversely, Bjordal and Johnson (2003) showed that the clinical results can be significantly improved if optimal parameters and dosage are used. However, only one of the studies in that review related to TKR. A systematic review of the TENS application on TKR concluded "that there is no utility for TENS in the post-operative management of pain after knee arthroplasty"[14]. As such, TENS is rarely used in this setting, despite there being a significant need for improved pain control for patients and a belief that the next biggest development in TKR may be a pain relief modality[15].

There are a number of factors that may contribute to the lack of efficacy of TENS in this application. Literature has shown that the variables of electrode placement[16-18], frequency of stimulation[19-21] and amplitude/waveform[22-24] all have an impact on clinical outcomes. Additionally, accommodation of nerves to stimulation during single treatments or following long-term use of an electrical modality has been reported[21]. In most electrical stimulation applications, the frequency and waveform component of stimulation has been the least understood and least manipulated parameter of stimulation. Electrical stimulation typically has been limited to setting sweeping frequencies, or establishing a ramping or random burst pattern while the amplitude of stimulation also plays a very important role. Even one parameter setting that is not optimally set may significantly reduce the efficacy of the treatment. Indeed, it is now understood that combining optimal treatment parameters can significantly improve clinical efficacy[22].

**Figure 1:**
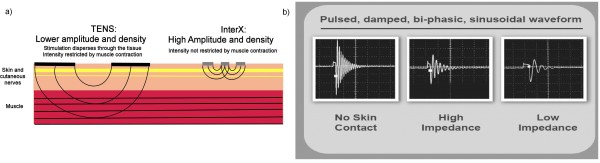
**A) Current Density B) Interactive Waveform **Figure 1a - Current amplitude and density: TENS compared to InterX. Figure 1b - Interactive waveform.

There is increasing evidence that successful optimization of electrode position, amplitude and frequency parameters in a dynamically changing pattern may well be the key critical to successful therapeutic outcomes[25-27]. The InterX is a device that provides such non-invasive interactive neurostimulation (NIN), optimizing all three stimulation parameters in a high amplitude, high density manner without penetrating too deeply into the tissue and without soliciting uncomfortable muscle contractions. The depth of penetration of electrical stimulation is proportional to both the electrode size and the distance between the electrodes. The closer electrodes are placed and the smaller the surface area, the shallower the depth of penetration[28]. If the stimulation does not reach the depth of the muscle, the amplitude is not restricted by muscle contraction. Thus, a device that delivers stimulation through an array of small, closely spaced electrodes will be capable of delivering higher amplitudes and therefore higher current densities than a device using larger electrodes[29]. This is a critical technological advancement of the InterX as research has demonstrated how important using higher amplitudes are to getting better clinical results[22,24]. In fact, it is current densities, which are the effective, measure of stimulus[30] in much the same way that pressure, not force, is the effective measure of manual therapy. Figure 1a shows a hypothetical illustration of the path of stimulation based upon known science when comparing two such models. The actual path of stimulation is determined by a number of factors and is hard to map precisely due to localised differences in tissue impedance[28]. The circuitry of the InterX allows the waveform to adjust as the impedance of the treatment area changes as the electrode is moved over the skin or in response to stimulation. The electrode does not require conductive gels, but is placed directly onto the skin surface. Because the human nervous system is adaptive, therapy sessions using the InterX rapidly vary the stimulation parameters interactively (Figure 1b) to prevent physiological accommodation, directing stimulation to optimal treatment points as well as protecting the skin from damage. These changes occur automatically as skin impedance changes in response to the electrical stimulation, which is caused by a combination of increased Galvanic Skin Response and electro-osmosis[28,31]. So there appears to be some scientific merit to claim by the manufacturers of InterX that the interactive technology supports a particular application that is aimed at optimizing parameters, which individually have been shown to elicit greater clinical benefit.

The InterX device operates by scanning the tissue to determine its impedance and to use the electrical characteristic of the skin to identify and target optimal treatment points. As stimulation is performed, the impedance of the skin under the electrode changes and is sensed by the device through the completed electrical circuit which in turn automatically varies the waveform parameters. Different preset stimulation patterns are selected in subsequent therapy sessions to prevent physiological accommodation and each of these presets automatically delivers a varying frequency to ensure optimal effects[20]. The presets in this study varied the frequency from 15-360 pulses per second using a mixture of burst, variable and amplitude modulated parameters.

**Figure 2:**
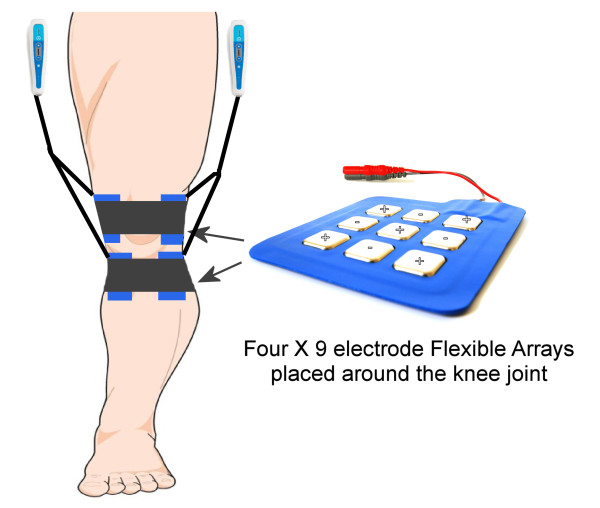
**Flexible array and device placement on operated leg**.

The handheld application of NIN has previously been shown to significantly reduce pain, medication intake and inflammation while also increasing range of motion following hip and ankle surgery[25,26]. In this study, the Flexible Array Electrode was used on post-operative TKR patients to treat their post-operative pain in combination with pharmaceutical analgesia and an aggressive rehabilitation protocol. This configuration of electrodes allows for tissue impedance scanning to be performed automatically. Each flexible array is comprised of nine electrodes; five of which are positive and four of which are negative. In this study four flexible arrays were used simultaneously which means 36 active electrodes (20 positive and 16 negative) were in contact with the skin around the knee(Figure 2). Ohms Law describes how electrical current will take the path of least resistance, so greater current density is delivered to these points of low impedance/resistance, as opposed to points of high impedance, by virtue of the electrode array configuration. The low impedance on the skin corresponds with myofascial trigger points and acupuncture points, which also have a high correlation with major nerve branches, and are treatment points which will respond best to electrical stimulation[32,33].

The objective of this investigation was to observe whether greater post-operative pain relief and range of motion (ROM) could be obtained by using the InterX 1000 device and Flexible Array Electrodes when combined with standard in-hospital rehabilitation protocols in patients undergoing primary TKR surgery as compared to standard in-hospital rehabilitation methods alone. Primary study endpoints (pain severity and knee range of motion (ROM)) would ideally show a reduction in pain severity and greater ROM in the InterX treatment group. Secondary endpoints (pain medication use and signs of inflammation) could result in a reduction in medication use and in knee joint inflammation. Circumferential measures of the knee were taken to try to demonstrate any possible changes in signs of inflammation. There is controversy about the reliability of this measure but we felt there was enough evidence to include this in the study as it has been used recently along with both skin temperature and inflammatory biomarkers in patients (other signs of inflammation that could not be realistically included in this study)[34,35].

## Methods

This randomised, controlled, prospective study was conducted under the Hywel Dda Clinical Audit and Risk Committee as it was performed to demonstrate the efficacy of a recognized technology on a new population of patients. The clinical audit passed ethics review through the Audit and Risk Committee. All subjects signed informed consent. The control group received the standard in-hospital rehabilitation protocol while the experimental group received standard in-hospital rehabilitation plus eight sessions of NIN therapy. Subjects were randomised to treatment group by the Theatre Operation List whereby subjects were randomly assigned treatment group by the order in which they were operated, alternating treatment group assignment in blocks of two.

61 subjects were enrolled into the study following elective total knee joint replacement from the orthopaedic clinic in the catchment area of Prince Philip Hospital, Carmarthenshire NHS Trust in Wales, UK. Surgery was performed using two main types of knee implant (cruciate retaining and cruciate sacrifice). Inclusion criteria specified that patients were to be 50-80 years of age with radiographic evidence of joint disease in at least 2 of the 3 knee compartments (including patellofemoral disease) who were scheduled for elective TKR. Subjects were to have no associated neurological deficit, sensory loss, paraesthesia, or hyporeflexia. Subjects were to have a clinically significant functional limitation (limited mobility and/or instability of the knee joint) and diminished quality of life prior to TKR. Additionally, subjects had to be willing to abide by the protocol and treatment schedule and sign informed consent. Subjects with local or systemic infections, medical conditions that substantially increase the risk of serious peri-operative complications or death were excluded from the study. Subjects with implanted neurostimulators, insulin pumps, and/or cardiac pacemakers were excluded due to the incompatibility of treatment with an electrical nerve stimulator. Subjects with epilepsy/seizure, dementia or cognitive disorders, pregnancy, psychiatric disease, active tumour or cancer, fracture or dislocation, or at substantial risk of venous thrombosis were also excluded from the study. Signs of venous thrombosis like redness and pain in the lower leg usually become evident within 72 hrs of surgery if they are going to occur. If the Physician saw any of these signs then treatment with the InterX was to be halted.

Subjects had standard operative anaesthesia to include general anaesthesia with either spinal anaesthesia or with femoral nerve block during the procedure. The nerve block was a single shot using morphine with doses ranging from 150-250 mcg. Post-operatively, pain was to be mediated from surgery to Day 2 by patient controlled analgesia (PCA) morphine (1 mg/mL) strictly monitored by a nurse, diclofenac suppositories (Volterol) and acetaminophen (Paracetamol). After 48 hrs, patients were stepped down to codeine with acetaminophen (Cocodamol) and diclofenac suppositories (Volterol) plus oral morphine for breakthrough pain. The medication was offered to patients and provided on an as needed basis (prn).

All subjects were followed for three days in-hospital prior to discharge to home. While in-hospital, the subjects underwent in-hospital rehabilitation exercises twice daily to include the following. On day one, subjects are transferred to a chair and try walking with a walking frame and they perform ROM exercises. On day two, subjects perform ROM exercises, walking with a frame and then walking with support using canes. On day three, subjects go to occupational therapy and do stair climbing, mimic getting in and out of a car, and walk with cane support. The goal is to get to 90° flexion.

Subjects randomised to the experimental group received NIN therapy according to the following schedule: Day 0 (0-24 hrs post-op), no treatment due to bandaging; Day 1 (24-48 hrs post-op), two InterX treatments of twenty minutes each using Preset 4 at approximately 1 pm and 4 pm; Day 2 (48-72 hrs post-op), three InterX treatments of 20 minutes each using Preset 4 (approx. 9 am; 1 pm and 4 pm); Day 3 (72-96 hrs post-op), three InterX treatments of 20 minutes each using Preset 1 (approx. 9 am; 1 pm and 4 pm). Baseline measures are those taken on Day 1 at approximately 1 pm (prior to NIN therapy for the experimental group). Final measures are those taken on Day 3 at approximately 4 pm prior to the final treatment.

NIN therapy was delivered via two InterX 1000™ devices, each with a Dual Flexible Array (Figure 2). Each array contains nine electrodes so a total of 36 electrodes are in contact with the skin around the knee. One pair of arrays was positioned just superior to the patella and one just inferior to the patella, with the electrode arrays positioned on the lateral and medial surfaces of the knee. One device delivered stimulation to the arrays on the medial side and the other device delivered stimulation to the arrays on the lateral side. The electrodes were directly in contact with the skin without conductive media. Preset 1 or 4 were used on the device and they cycled through various parameters for the duration of the 20-minute application. Preset 1 delivered 30-120 pulses per second (PPS); 15-60 PPS and 15 PPS. Preset 4 delivered 90-360 PPS in a variable burst pattern; 30-120 PPS; 240 PPS in a burst pattern and 3:1 amplitude modulation at 120 PPS. The amplitude was increased by the nurse practitioner to a level that was strong but comfortable tingling to the patient and all four arrays were used at the same time.

In the initial version of the protocol, the study measurements were assessed at each time point. However, because of the time involved in study measurement collection imposed on the nursing/rehabilitation routines, the protocol was amended to collect ROM and knee circumference data only at the first (Baseline) and last (Final) study time period. VRS was recorded at every treatment and medication was recorded as taken. The primary study variables were designated as the VRS and ROM; secondary study measures included the total daily pain medication taken and knee circumference. Study measurements were taken at 9 am, 1 pm and 4 pm for the control group, and just prior to and following NIN therapy administration at those same times for the experimental group.

### Verbal Rating Scale

Subjects verbally rated the intensity of their knee pain using an 11-point numeric scale (0 = "no pain"; 10 = "worst pain possible"). This scale, similar to the Visual Analogue Scale (VAS), has demonstrated validity and reliability[36]. At Baseline, all subjects were asked to rate the intensity of their current pain with the knee in active flexion. Prior to and following each application of InterX, experimental group subjects rated their current pain with the knee in active flexion. Patients were encouraged to flex their knee as far as they could. Control subjects had only a single measurement taken twice on the first day, and three times per day thereafter at approximately the same time of day as the experimental group was measured.

### Range of Motion

Knee flexion was measured by the nurse using a standard goniometer (20 cm Jamar-E-Z by Physiomed). At Baseline and Final, all subjects were measured. Experimental group subjects were measured pre- and post-treatment at each time point. Control subjects had only a single measurement at each time point. Patients were seated and asked to actively flex their knee as far as pain would allow. This flexion was then measured by the nurse practitioner.

### Medication Log

The dose and frequency of all pain medications were recorded daily for all patients in mg for PCA morphine use and for diclofenac suppositories, and pill counts for acetaminophen, and codeine with acetaminophen using standardized dosage forms.

### Inflammation/Oedema

The circumferential girth at 2 inches above the knee of both the affected and unaffected knees was measured. The un-operated knee measurement was subtracted from the operated knee measurement as a possible indicator of signs of inflammation of the operated knee. These measures were made at Baseline and Final time points for all patients.

#### Sample Size Calculation

The trial's sample size was determined based on observed variation in pain scores from earlier studies, and the investigators' judgment that a two-point reduction in reported pain (on a scale of 1 to 10) represented a clinically meaningful result. It is generally accepted that a 30% reduction of pain in acute cases represents a clinically meaningful change[37], so a two-point reduction applies to this population of patients who typically average between 4 and 6 out of 10 on the pain scale following surgery. The study was designed to provide power of 80% with an alpha level of 0.05, and allowed for 20% loss to follow-up. Therefore, 30 subjects/group, 60 subjects total, were to be enrolled into the study.

#### Demographics

**Figure 3:**
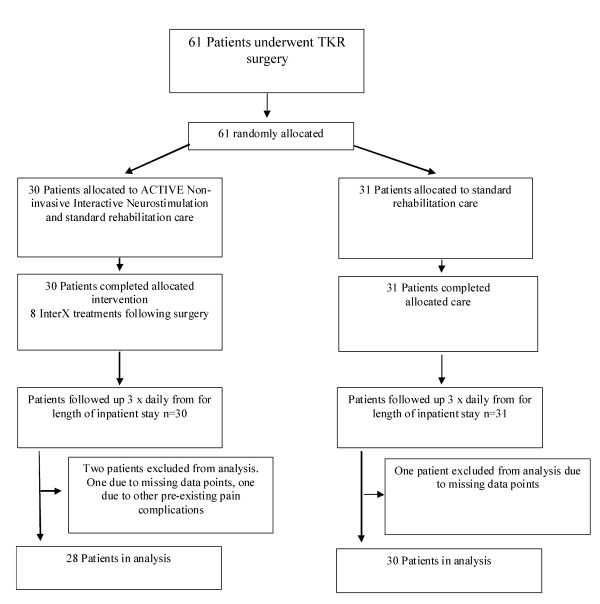
**CONSORT Chart**.

Sixty-one subjects signed informed consent and were enrolled into the study. Two subjects, one in each treatment group, were excluded from the analysis due to missing data from either the Baseline or the Final time point so that calculations regarding differences from Baseline to Final could not be determined. Additionally, one subject in the InterX group was determined to have a pre-existing condition of rheumatoid arthritis that confounded the data analysis and so she was excluded *post-hoc*. Ultimately, 28 subjects in the experimental group and 30 subjects in the control group are included in the below analyses (Figure 3).

Table 1 shows the demographic and baseline characteristics of both treatment groups. There was no significant difference between groups with regard to gender or age. However, a clinically significant difference between groups was noted in other parameters. The experimental group had an average pain score, which was 1.5 points higher than the control group, bordering on the difference required between groups for the power calculation. Additionally, there was a statistically significant difference between the two groups in the baseline ROM measurement (p = 0.0005), with a mean ROM for the InterX group of 45.7 and a mean ROM for the control group of 60.4. The standard error of the estimated difference between the two groups (15.8) was 4.13 Surprisingly, the difference between the circumferences of the affected and un-affected knees was 1.2 cm less in the InterX group at Baseline than the control (p = 0.03).

**Table 1:** Patient demographics and baseline characteristics

	Control Group N = 30	InterX Group N = 28
Gender		
Male	15	15
Female	15	13

Average Age (years)	69.3	67.9

Baseline* VRS	4.4	5.9

ROM (degrees)		
Pre-op	109.5	110.4
Baseline	60.4	45.7

Baseline* Inflammation (cm)	4.7	3.5

*Baseline here is defined as the first measurement on Day 1 after the bandages have been removed.

The groups are clinically significantly different in three variables: Pain (VRS) was higher in the InterX group; ROM was poorer in the InterX group; inflammation/oedema was higher in the control group.

## Results

### Verbal Rating Scale (VRS) Pain Scores

**Figure 4:**
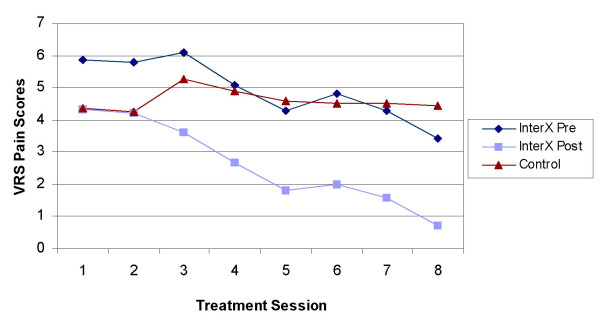
**VRS pain scores during joint mobilisation**.

The effect of NIN therapy with the InterX as compared to control on the change in VRS between the Baseline and the Final study time periods is presented in (Figure 4). For the experimental group, the "before therapy" scores were used in both instances as this seemed to reflect a long-lasting, cumulative effect of NIN therapy vs. a potentially transient effect following therapy. An analysis of covariance (ANCOVA) was used with the Baseline VRS score serving as the covariate term. The treatment effect was highly significant (p = 0.002), with an adjusted mean change for the InterX group of -2.15 improvement vs. an adjusted mean change of improvement for the control group of -0.34. The standard error of the estimated difference between the two groups (1.81) was 0.57.

**Figure 5:**
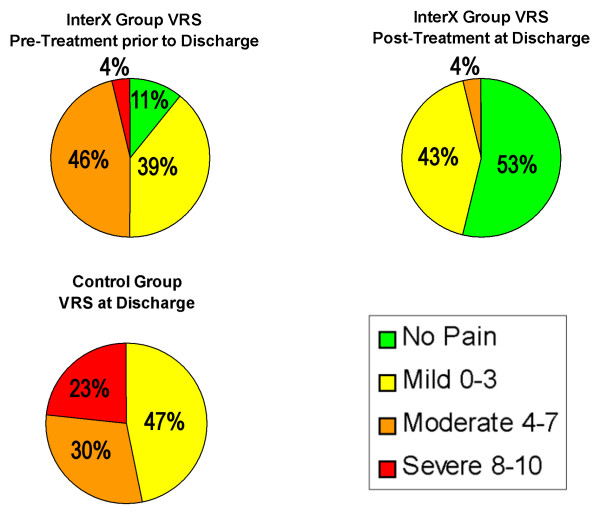
**Distribution of VRS patient pain scores during flexion at Final time point**. The chart shows the level of pain for each patient in the Control group and in the InterX group at both pre-NIN therapy and at post-NIN therapy. At Final post-NIN therapy, 27 of 28 patient had only Mild or No pain (0-3, VRS).

In Figure 5, the patients' pain scores at the Final time point were plotted. 3 subjects (11%) in the InterX pre-NIN therapy group reported 0 or no pain; 11 subjects (39%) with mild pain (VRS = 1-3); 13 subjects(46%) reported moderate pain (VRS = 4-7); 1 subject (4%) reported severe pain (VRS 8-10). The control group had no patients with zero pain scores, 14 subjects(47%) with mild pain; 9(30%), with moderate pain; and 7 (23%) with severe pain (VRS = 8-10) at Final. The InterX post-NIN therapy group had 27 of 28 subjects (96%) with none or mild pain vs. the control group with a total of 14 subjects(47%) reporting only mild pain and no patients reporting being pain free. Overall, at the Final pre-treatment measure, only three patients in the InterX group had a higher pain score than at Baseline compared to thirteen control patients. Post-treatment immediately prior to discharge, all InterX patients reported a lower pain score than that Baseline.

**Figure 6:**
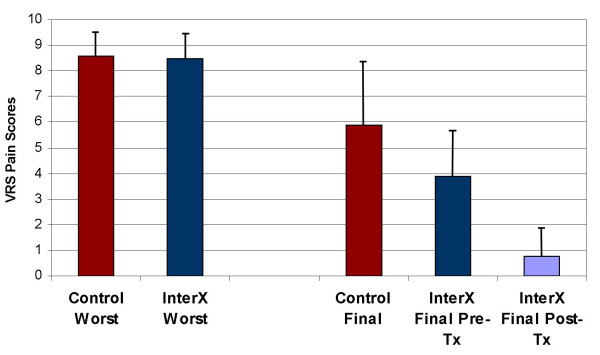
**Change in VRS pain scores for severe pain sub-groups (VRS > 6)**. Error bars are Standard Deviation

A similar analysis was conducted on a subset of "high pain" patients identified by selecting individuals with a recorded VRS of 7 or greater for any time period (Figure 6). The treatment effect for this subset of patients was highly significant (p = 0.006), with an adjusted mean pain reduction for the experimental group of 2.66 point vs. an adjusted mean pain reduction for the control group of 0.49. The difference between the two groups was 2.18 (S.E. 0.73). The average worst pain during the study was 8.4 and 8.5 in the NIN and control severe pain patients respectively. At discharge, the control group reported a VRS of 5.9 while the experimental group reported an average of 3.9 before the last NIN treatment. Following the last treatment, the average VRS in the experimental group during knee flexion was 0.8, which was immediately prior to discharge.

#### ROM

**Figure 7:**
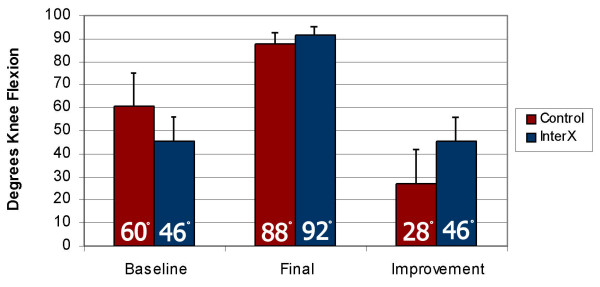
**Increase in range of motion during rehabilitation**. Error bars are Standard Deviation. Figures rounded to nearest degree.

The effect of treatment (NIN vs. control) was analysed on the change in ROM between Baseline and Final (Figure 7). An analysis of covariance was used with the pre-surgery ROM serving as the covariate term. We found the covariate term was not significant, so it was dropped from the final model. The treatment effect was highly significant, with a mean improvement in ROM for the experimental group of 45.7° vs. a mean improvement for the control group of 27.2°. The estimated difference between the improvement in each group was 18.4° (S.E. 4.29) with the experimental group showing significantly greater improvement than control (p = 0.0001). The goal of rehabilitation was to get the subject to 90° flexion prior to discharge. Both treatment groups met this goal, but the experimental group had a greater deficit to regain to get there in the same length of time.

#### Medications

Table 2 shows the daily medication use that was tracked during the study for PCA, diclofenac suppositories, acetaminophen, codeine with acetaminophen and oral morphine. The oral morphine data was converted to the equivalent PCA dosage (3:1 ratio of analgesic effectiveness)[38] and added to the PCA data. Total medication use was compared between groups. Both parametric (Student's t-test) and non-parametric (Mann-Whitney test) methods were used to individually analyse the effect of treatment (InterX vs. control) on total consumption of each of four medications. P-values for the parametric and non-parametric tests were non-significant for all drugs, indicating that there was not a statistically significant decrease in medication though there may be clinically significant implications for patients taking 9% less morphine, nearly 30% less NSAIDs and 10% less cocodamol. A multivariate test of differences (MANOVA) between the two treatment groups for all four medications was performed. The treatment effect was not significant (p = 0.64) using the Wilks Lambda statistic.

**Table 2:** Patient medication intake

Medication Intake	Control	InterX	% Difference	P value
**PCA** (mg/day)	32.73	29.7	9.3%	p = 0.68
**Voltarol** (100 mg/day)	315	230	27%	p = 0.28
**Paracetamol** (# 500 mg pills/day)	7.23	7.1	1.8%	p = 0.90
**Cocodamol** 30/500/per day	4.47	4.0	10.5%	p = 0.56

In retrospect, it is believed that this measure in this situation was probably an inappropriate measure to include because the goal of post-operative pain medications in a procedure of this magnitude, is to stay ahead of the pain and so standard administration of pain medicines at regular intervals are administered by hospital staff regardless of the subject's level of pain. It is noteworthy that the experimental group, who started with higher levels of pain at Baseline, reported lower levels of pain, or no pain, using the same amount of pain medications as the control group who were reporting no significant relief from pain. It was deemed not possible to convert the different types of medications into one measure as they included opioids and NSAIDs. So this data stands as a backdrop to demonstrate that the pain reduction seen in the experimental group was due to the NIN treatment and not due any variations in medication intake. It should be noted that the experimental group did take less medication than the control (see Table 2), but this failed to show statistical significance.

#### Inflammation/Oedema

The effect of treatment (NIN vs. control) was analysed on the change in inflammation (measured as the difference between circumferences of the non-operated and operated knees) between the Baseline and Final. An analysis of covariance was used with the inflammation measured during the Baseline serving as the covariate term. The treatment effect was not significant (p = 0.44), with an adjusted mean change for the experimental group of 1.11 cm vs. an adjusted mean change for the control group of 1.59 cm. The standard error of the estimated difference between the two groups (0.48) was 0.67. Note that both groups experienced an increase in the circumference of the affected knee following the surgery as is normal for this type of invasive procedure. The difference between treatment groups at Final is consistent with the difference seen at Baseline. However, it should be noted that the experimental group took on average 27% less NSAIDs. The average change in knee circumference was very small and almost within the normal variations expected with repeated measures, which may indicate that this circumferential method in this study may not have been a reliable way to measure changes in inflammation[39]. So while the experimental group were discharged with significantly less circumferential differences between the affected and unaffected knees (p < 0.05), it is impossible to draw any conclusion from this data point due to the complexity of this outcome and the variations in NSAID intake between the groups.

## Discussion

This study was undertaken as an audit of the Hywel Dda NHS Trust in Wales to validate the clinical benefit of NIN therapy as a supplemental rehabilitative therapy following TKR. The hospital currently uses NIN therapy as a standard course of therapy in the chronic pain clinic. The results of this study clearly demonstrated the clinical benefit of NIN therapy with the InterX device as a supplement to the standard in-house rehabilitative protocol for patients following TKR. By random assignment to treatment group, subjects with more severe pain and more ROM-restricted were placed in the InterX group. Within a relatively short 3-day period of time, patients in the InterX group obtained the necessary ROM for discharge and did it experiencing significantly reduced levels of pain compared to those in the control group.

In making conclusions regarding the effectiveness of electrical stimulation to counteract pain, several relevant ideas have been discussed by Bjordal [22] in the meta-analysis of TENS used to reduce post-operative analgesic consumption. First, is the idea that TENS is effective *only *if it used optimally and that amplitude and frequency appear to be the most important variables. Secondly, that TENS is only effective for partial pain relief whereas analgesic pharmaceuticals have the potential for complete pain relief, though side effects often mitigate this. Typically, TENS has been considered effective only if the supplemental use of TENS results in a reduction of analgesic consumption without increased pain scores. In Bjordal's article, it is suggested that a positive outcome also occurs when there is a decrease in pain scores, but the analgesic consumption (at a tolerable level) is maintained. The author also mentions that the best treatment effects were observed using the higher frequencies of TENS at 85 Hz and at an amplitude of > 15 mA or a strong sensation to the patient.

Within this study, the latter scenario was observed. Subjects here experienced greater pain relief on tolerable pain medication regimens. The greater pain relief seen in this study allowed the patients to push harder in rehabilitation to reach the discharge criteria since the InterX treatment group was clinically more severely hampered with pain and ROM restrictions at Baseline. It should be noted that the InterX was used at a variable frequency covering the optimal range suggested by Bjordal et al and also at an amplitude in the range of > 40 mA[29]. It is of interest, that after the study was complete some of the severe patients in the control group received NIN therapy to bring their pain down so that they could engage in the rehabilitation process. Medication alone was not sufficient, without inhibitory side effects, to reduce pain for these patients.

Evidence shows that InterX NIN therapy probably activates the gate control mechanism of pain control, but due to the short treatments, long-lasting effects and cumulative reductions in pain reported by patients, gate control probably is not the primary mechanism of pain relief[25-27]. InterX NIN therapy targets low impedance areas of the body (trigger points)[40]. It is believed that increases in blood flow and sweat secretion account for the changes in skin impedance[40]. As the skin is stimulated, its impedance changes[28]. The InterX rapidly modifies the waveform and amplitude in response to these impedance changes which allows for a significantly greater concentration (current density) of stimulation without risking harm to the skin. It is hypothesized that the delivery of high amplitude stimulation to multiple treatment points in this way enhances the pain relief achieved as compared to lower amplitude stimulation delivered only over the pain site. The mechanism of pain relief for this type of cutaneous stimulation is suggested to include both segmental and descending inhibition[23]. More recently it has been shown that NIN therapy activates significantly greater production of certain cytokines and genes than TENS which may optimize the inflammatory process and reduce pain. NIN stimulates the production of Adenosine Triphosphate(ATP)[41] which would have both an anti-inflammatory effect as well as reduce pain segmentally[42]. The inability of TENS technology to deliver such high current densities so specifically may explain why TENS clinical studies in this application have been unsuccessful in the past[14]. By reducing pain during movement prior to a rehabilitation session the patient may be able to perform exercises with greater ease, work harder, and have less discomfort while exercising which has potential benefits for a population of patients who need to return to function as quickly as possible[15].

## Conclusions

The results of this study clearly demonstrated that the delivery of NIN through the Flexible Array Electrode is effective for the management of pain in this population of patients, supporting previous literature pertaining to the handheld application of NIN in the post-surgical setting. The InterX is designed to optimize treatment point location, amplitude and frequency to ensure better clinical results and this technological aspect is maintained in this application. The clinical benefit of NIN therapy with the InterX device as a supplement to the standard in-house rehabilitative protocol allows patients suffering pain to regain function quicker, especially if their pain levels are particularly high. Even though subjects with more severe pain and more ROM-restriction were randomly assigned to the InterX group, the subjects receiving NIN therapy with InterX fared much better clinically with significantly reduced pain levels and improvements in ROM compared to control subjects. The implications of these findings are that patients suffering severe pain following TKR struggle to get sufficient relief from the standard of care of medications and that the inclusion of NIN therapy into the standard of care will offer greater and more consistent pain control, without the need for increased medication even with the worst cases.

## List of Abbreviations

ANCOVA: analysis of Covariance; cm: centimetres; MANOVA: multivariate test of differences; NSAIDS: nonsteroidal anti-inflammatory drugs; NIN therapy: non-invasive interactive neurostimulation; PCA: patient controlled analgesia; ROM: range of motion; TENS: transcutaneous electrical nerve stimulator; TKR: total knee replacement; VRS: verbal rating scale

## Competing interests

The author(s) AN and DT declare that they have no competing interests. Author ZV is a paid consultant for Neuro Resource Group and holds shares in the company, although she was not involved in collection or interpretation of data.

## Authors' contributions

AN was the principle investigator and oversaw the implementation of the protocol. DT performed all treatments, measurements and data collection. ZV assisted in the development of the study protocol only. All authors have read and approved the final publication article.
